# Supplementary material for: Desert Endemic Plants in Algeria: A Review on Traditional Uses, Phytochemistry, Polyphenolic Compounds and Pharmacological Activities
Source: Molecules. 2023 Feb 15;28(4):1834. doi: 10.3390/molecules28041834 (PMC9959599; doi:10.3390/molecules28041834)
Supplement: Supplementary file 1 [file molecules-28-01834-s001.zip › molecules-2130606-supplementary.pdf]

molecules

A review

Desert Endemic Plants in Algeria: A Review on Traditional Uses, Phytochemistry, Polyphenolic Compounds and Pharmacological Activities

Hadia Hemmami[1,2], Bachir Ben Seghir[1,2,3], Soumeia Zeghoud[1,2], Ilham Ben Amor[1,2], Imane Kouadri [2,3,4], Abdelkrim Rebiai[2,5], Abdelmalek Zaater[6,7], Mohammed Messaoudi[5,8,], Naima Benchikha[5], and Barbara Sawicka[10] and Maria Atanassova[10,*]

Figure S1. Medicinal plant used in Saharan regions.

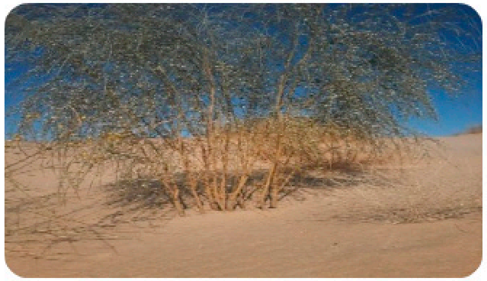

Retama Retam Web b

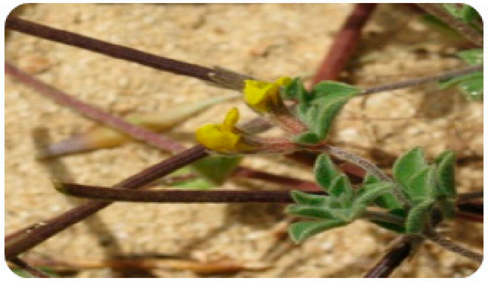

Lotus halophylus Boiss

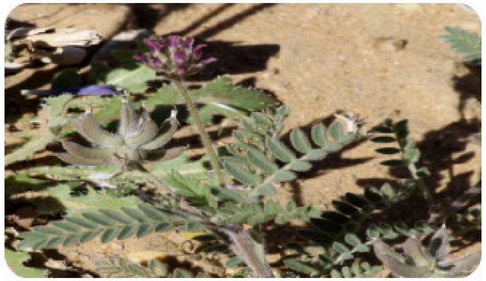

Astragalus cruciatus Link

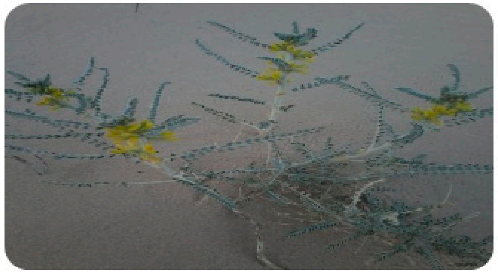

Astragalus gombiformis Bomel.

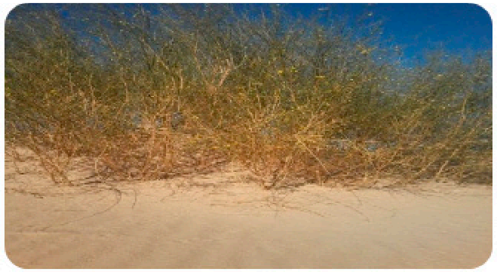

Genista saharae Cosson et Dur.

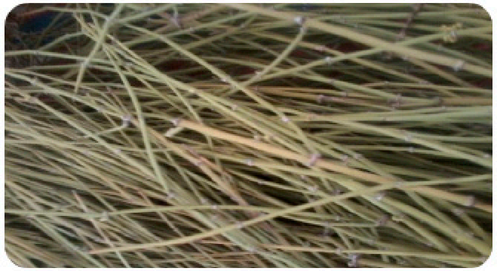

Ephedra alata DC.

molecules
MDPI

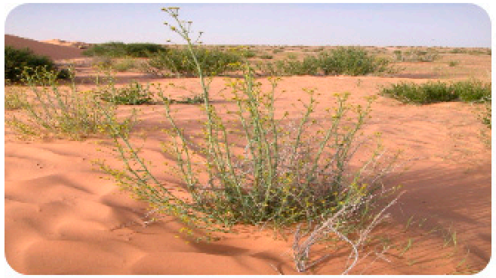

Eurphorbia guyoniana Bois et Reut.

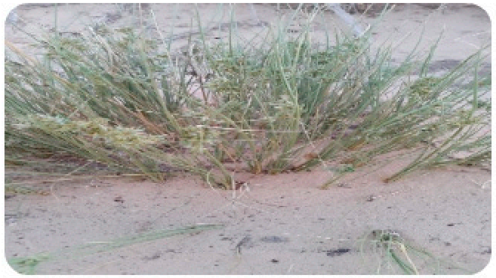

Cyperus conglomerates

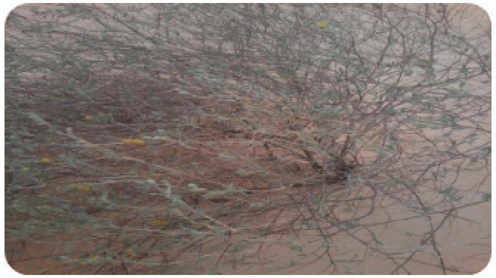

Heliathemum lipii (L.) Pess

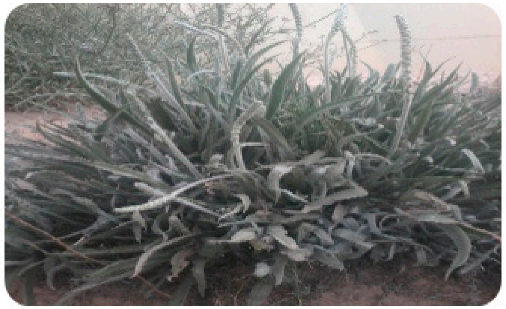

Plantago albicans L.

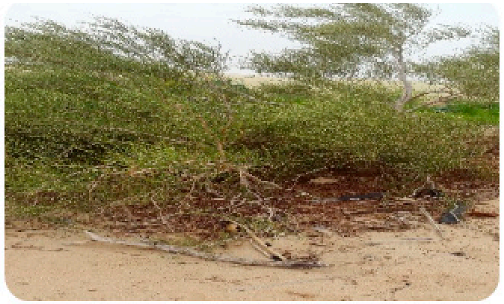

Calligonum comosum L'her.

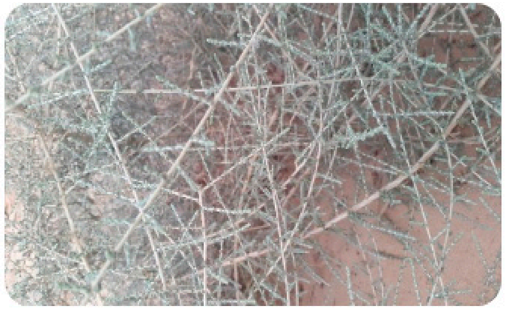

Tamarix boveana

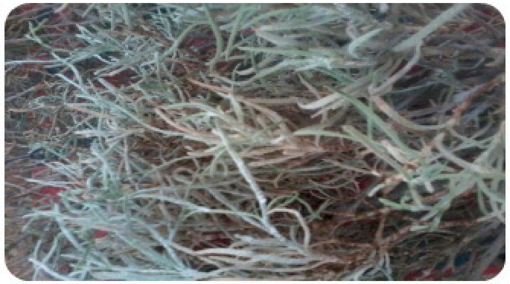

Limoniastrum guyonianum Dur.

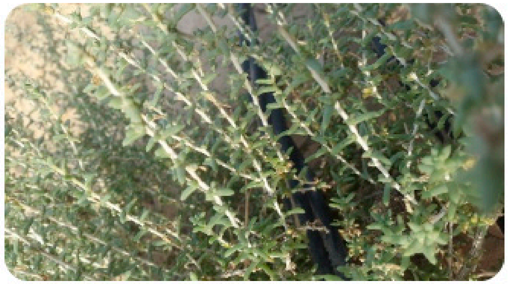

Traganum nudatum Del.

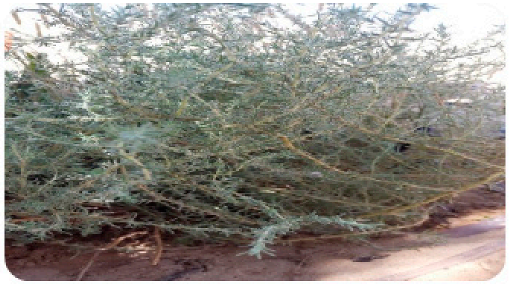

Bassia muricata (L.)

molecules

MDPI

Table S1. Main biological activities of Plants in Saharan regions, part of the plant that was analysed, extract type, test system, and effects are summarized.

Name of the plant	biological Activity	Extracts	Test systems	Effects	Study	Dosage	Plant part	bioactive compounds	Ref
Retama Retam Web b	Analgesic, Antioxidant Activities	Flavanol isolated from Methanol ; Methanol/ Water (5:1)	DPPH	16.70 ± 5.28	*In vitro*	1 mg/kg	Aerial parts	Genistein , 6-hydroxygenistein, pratensein (4' methylorobol),	[169]
	Antioxidant, antimicrobial	Diethyl ether	DPPH ; both disc diffusion and dilution methods	Active	*In vitro*	0.1692 µg/mL	Leaves and seeds	Flavonoids	[170]
Lotus halophylus Boiss	Antimicrobial activity	Aqueous, ethanol and butanol	both disc diffusion and dilution methods	Active	*In vitro*	2000 µg/disc	Aerial parts	Different chemical compounds	[171]
Genista saharae Cosson et Dur.	Antioxidant activities	Methanol	DPPH	Active	*In vitro*	IC50= 0.247 mg/ml	Aerial parts	Phenolic compounds	[172]
Astragalus gombiformis Bomel.	anti - diabetic, an tioxidants and anti - Alzheimer's dis ease	Butanolic fractions	DPPH, ABTS GOR, pha - amylase in hibitory anti – Alzheimer Activities	Active	*In vitro*	Active	Stems, flowers, leaves, pods and seeds	Total phenolic content Total flavonoids content	[173]

	Anticholinesterase activity	Petroleum Ether, Dichloromethane, and Methanol	Colorimetric method	Active	*In vitro*	IC50 of 110 µg/ml.	Leaves	Undefined	[174]
Eurphorbia guyoniana Bois et Reut.	Toxicity Study	Acetone	Desert locusts	Active	*In vivo*	Active	Leaves	Undefined	[175]
	Antibacterial effect	Water	Both disc diffusion and dilution methods	Active	*In vivo*	Active	Aerial parts	Undefined	[176]
Ephedra alata DC.	Antioxidant, analgesic and hypolipidemic effects	Methanol	DPPH Haemolysis assay	Active	*In vitro*	Active	Aerial parts	Polyphenolic compounds	[177]
	Free-Radical-Scavenging, Antibacterial	Hexane, ethyl acetate, and methanol	DPPH	Active	*In vitro*	Active	Aerial parts	Essential Oil	[178]
Heliathemum lipii (L.) Pers.	Antioxidant	Methanol	DPPH-TAC- OH•-RP	Active	*In vitro*	Active	Leaf, stem-fruit	Polyphenolic compounds	[179]

	Effect on the smooth muscle	Ethyl acetate n-butanol	Ested on the rat distal colon	Active	*In vivo*	Active	Aerial parts	polyphenolic compounds	[159]
Cyperus conglomeratus	Anticandidal activity	Water	Both disc diffusion	Active	*In vivo*	Active	Aerial parts	Essential Oils	[180]
	Antiulcer Potential	Methanol (7 0%)	Rats	Active	*In vivo*	25, 50, 100 mg/kg	Aerial parts	Flavonoids, stilbenes, aurones, quinones, terpenes, and steroids	[181]
Calligonum comosum L'her.	Anti-fungal potential	Ethanol	Alternaria spp and rhizopus spp	Inhibited the fungal growth	*In vitro*	3.13 and 12.50 mg/ml	Leaves, stem and roots	Undefined	[182]
	Analgesic activity	Ethanol, Methanol	Writhing test	Active	*In vitro*	Active	Aerial parts	Polyphenolic compounds	[183]
Plantago albicans L.	Cytogenetic effects	Water	Cytogenetic assay	Active	*In vitro*	Active	Root leaves	Undefined	[184]
	Beneficial effects	Ethanol	Rats	Active	*In vivo*	Active	Leaves	Polyphenolic compounds	[76]
Limoniastrum guyonianum Dur.	Acute Heamatotoxicity	Water	Haematological Analysis	Active	*In vivo*	Active	Leaves	Undefined	[185]
	Antioxidant activity	Methanol, Ethanol	DPPH, ABTS	Active	*Invitro*	Active	Aerial part	Polyphenolic compounds	[186,18 7]
Tamarix boveana	Antimicrobial activity	Water	Both disc diffusion and dilution methods	Active	*In vitro*	Active	Aerial parts	Volatile compounds	[188]

	Antifungal activity			Not active	*In vitro*	Not active			
Traganum nudatum Del.	Antioxidant activity	Ethyl acetate	DPPH	Active	*In vitro*	Active	Aerial parts	Polyphenolic compound	[189]
	Antimicrobial activity	Water	Both Disc diffusion	Active	*In vitro*	Active	Aerial parts	Polyphenolic compound	[190]
Bassia muricata (L.)	Antioxidant activity, and allelopathic effect	Water	DPPH ABTS	Active	*In vitro*	Active	Aerial part	Essential oil	[191]
	Antibacterial and antioxidant activities	Ethanol	DPPH Both disc diffusion and dilution methods	Active	*In vitro*	Active	Aerial part	Polyphenolic compound	[192]
Atriplex halimus L.	Antimicrobial effects	Water	Both disc diffusion and dilution methods	Active	*In vitro*	13.5 and 14 mm	Aerial part	Polyphenolic compound	[193]
	Antidiabetic activity	Water	Toxicity evaluation in rat	Active	*In vivo*	Active	Leaf	Polyphenolic compound	[86]
Zygophyllum album L.	Antioxidant	Methanol	DPPH-TAC- OH°-RP	Active	*In vitro*	Active	Stem	Total polyphenols and flavonoids	[194]
Matricaria pubescens (desf) Schultz.	Antihypertensive Activity	Water	Male rats	Active	*In vivo*	Active	Aerial parts	Total polyphenols	[195]
	Antioxidant activity	Water	FRAP	Active	*In vitro*	Active	Aerial parts	Total polyphenols	[196]
Launaea resedifolia O. K.	Antioxidant capacity	Ethyl acetate, water	DPPH , RP,	Active	*In vitro*	Active	Aerial parts	Total polyphenols and flavonoids	[197]

molecules

MDPI

	Antimicrobial Activity	n-Butanol	Both disc diffusion	Active	*In vitro*	Active	Aerial parts	Flavonoids	[198]
Solanum nigrum L.	Antiviral Activities	Methanol	SINV, Sindbis virus; HSV, Herpes Simplex virus; Polio, polio virus	Active	*In vitro*	6.5mg/ ml,	Fruits	Different chemical Compounds	[199]
	Antimicrobial activity	Methanol	Both disc diffusion and dilution methods	Active	*In vitro*	Active	Leaves	Undefined	[200]
Erodium glaucophyllum L′Her.	Antioxidant	Methanol/ Water (80:20, v/v)	DPPH (FRAP) ABTS•	Active	*In vitro*	Active	Leaves and flowers	Different chemical Compounds	[166]
	Antimicrobial		Both disc diffusion and dilution methods						
Cleome arabica L.	Antioxidant	Methanol	DPPH•	Active	*In vitro*	Active	Aerial parts	Total polyphenols and flavonoids	[201]
	Antimicrobial	Water	both disc diffusion	Active	*In vitro*	Active			
Neurada procumbens L.	Antioxidant potential	Methanol/ Water (80:20, v/v)	DPPH, catalase antioxidant activity (CAT) and ABTS	Active	*In vitro*	Active	Aerial parts	Total polyphenols and flavonoids	[202]
	Cytotoxic activity		MTT assay						
